# A model of visual limitation in patients with keratoconus

**DOI:** 10.1038/s41598-020-76489-1

**Published:** 2020-11-09

**Authors:** Antonio Pérez-Rueda, Gracia Castro-Luna

**Affiliations:** 1 Ophthalmology Department, Torrecárdenas University Hospital, Almería, Spain; 2 University of Almería, Almería, Spain

**Keywords:** Diseases, Eye diseases, Corneal diseases

## Abstract

This paper aims to calculate a relevance model of visual limitation (V.L.) in keratoconus patients based on refractive and topographic parameters. A cross-sectional study was carried out in Torrecárdenas Hospital, Almería, Spain, between February 2018 and July 2019. It included 250 keratoconus patients. Two groups were created according to a grading system of V.L. based on RETICS (Red Temática de Investigación Cooperativa en Salud) classification: keratoconus patients with no V.L. (best spectacle-corrected visual acuity (BSCVA) ≤ 0.05 logMAR) and keratoconus patients with V.L. (BSCVA > 0.05 logMAR). Correlations and a binary logistic regression were established. V.L. was correlated with maximum curvature (*r* = 0.649, p < 0.001) and root mean square higher-order aberrations (HOARMS) (*r* = 0.625, p < 0.001). Binary logistic regression included V.L. as the dependent variable and spherical equivalent, HOARMS, spherical aberration and interaction between the anterior and posterior vertical coma as independent variables. The model was a good fit. Area under the curve (A.U.C.) of receiver operating characteristic (R.O.C.) curve was 0.924, sensitivity 91.90%, specificity 83.60%, accuracy 88.94%; and precision 91.17%. Binary logistic regression model of V.L. is a good fit model to predict the early loss of visual acuity in keratoconus patients.

## Introduction

Keratoconus is an asymmetric bilateral eye disease^1^ with corneal thinning and protrusion that occurs in a cone form, generally lower and temporal^2–4^. This corneal deformation produces a significant decrease in visual quality because of irregular astigmatism and higher-order aberrations^5,6^. It usually appears in adolescence, progressing into the third or fourth decade. The beginning in youth could influence people’s learning and their quality of life. Although unknown etiology, it has been related to genetic^5^ and environmental factors^7–9^. Keratoconus incidence and prevalence are very variable. Recently, Bak-Nielsen et al*.*^10^ have published the average incidence rate 2011–2015 was 3.60 per 100,000 person-years, and the prevalence of diagnosed keratoconus in the Denmark National Patient Register 1977–2015 was 44 per 100,000 persons. Values fivefold to tenfold higher than previously were reported by Godefrooij et al*.*^11^ who published annual incidence of keratoconus was 13.3 per 100,000 person-years, and the estimated prevalence of keratoconus was 265 cases per 100,000 persons in the Netherlands.

The diagnosis of keratoconus is essentiality clinical. Subclinical keratoconus (SKC), is defined to an eye with topographic signs of keratoconus and suspicious topographic findings (mild asymmetric bow-tie with or without skewed axis and low anterior curvature) under slit-lap examination and keratoconus in the fellow eye^12^.

Several classifications of clinical keratoconus have been used, for example, the Amsler–Krumeich classification^13,14^. However, most of them have classified the topographical characteristics of the keratoconus but not the limitation of the visual function of these patients. Alió et al.^15^ published in 2011 a grading system by the level of V.L. based on RETICS (Red Temática de Investigación Cooperativa en Salud) classification. It is a functional scale based primarily on best spectacle-corrected visual acuity (BSCVA)^15–17^.

Different methods can measure the visual function, and it is the result of integrating the function of different ocular structures, not only the cornea. Uncorrected visual acuity (UCVA) and best spectacle-corrected visual acuity (BSCVA) in Snellen scale (6/6 m or 20/20 fits, visual acuity = distance at which test is made/distance at which the smallest optotype identified subtends an angle of five arcminutes) or logMAR (Logarithm of the Minimum Angle of Resolution) are different ways of measuring it. A logMAR chart, also called an ETDRS chart (Early Treatment Diabetic Retinopathy Study), is an useful chart with letters lines to measure visual acuity. When using a LogMAR chart, visual acuity is calculated with reference to the logarithm of the minimum angle of resolution.

Corneal topography is a non-invasive diagnostic test useful to know several corneal characteristics^18,19^. The Oculus Pentacam system provides the anterior and posterior topographic, pachymetry and aberrometry maps^20^. Topography is essential for the study of keratoconus, and its variables can be determinant of the patient visual acuity. The quantity and quality of vision are correlated with topographic indices^21,22^. Several studies have demonstrated the relationship between severe V.L. and different variables such as spherical equivalent, mean keratometry, asphericity, intraocular pressure, corneal resistance factor and several higher-order aberrations^15–17^.

The main objective of this study is to establish a predictive model of V.L. in patients with keratoconus based on topographic variables obtained with Pentacam.

## Methods

### Patient selection and study design

A cross-sectional study was carried out to analyse the topographic, pachymetric and aberrometry variables obtained using a rotating Scheimpflug camera (Pentacam) from patients with keratoconus in the Department of Ophthalmology at the Torrecárdenas Hospital, Almería, Spain between February 2018 and July 2019. These were collected from the Pentacam clinical database. All experiments were performed following relevant guidelines and regulations. All experimental protocols were approved by Almeria Research Ethics institutional and licensing Committee (C.E.I./CEIm) located at Torrecárdenas Hospital with the following committee’s reference number: 19/2019. Participants were previously informed of the data to be taken and signed an informed consent authorizing the use of their data anonymously. The ethical principles for medical research on human beings of the Declaration of Helsinki were followed.

Two hundred and fifty eyes of 250 patients with keratoconus were distributed in the different stages of keratoconus:

Forty eyes with SKC. This early-stage included patients with (1) Slight topographic signs of keratoconus and suspicious topographic findings (mild asymmetric bow-tie with or without skewed axis) (2) Mean K (mean curvature of keratometry) < 46.5 D (3) minimum corneal thickness (MCT) > 490 μm (4) no findings in the slit lamp (no central thinning with Fleischer's ring nor Vogt's striae) (5) clinical keratoconus in the fellow eye.

Two hundred and ten patients with keratoconus. Every eye with keratoconus presented with at least one biomicroscopic alteration of the anterior segment (central thinning with Fleischer's ring and Vogt's striae) and topography compatible with corneal ectasia. In patients with bilateral keratoconus, one of the eyes was taken randomly. Physician made the decision choosing a random number from two, one for each eye. They were categorized in grades according to Amsler–Krumeich classification: 122 eyes with grade I (eccentric corneal protrusion, induced astigmatism < 5D, mean K < 48 D), 55 eyes with grade II (induced astigmatism 5D – 8D, mean K < 53 D, absence central scars, MCT > 400 μm), 19 eyes with grade III (induced astigmatism 8D–10D, mean K < 55 D, absence central scars, MCT between 300 and 400 μm) and 14 eyes with grade IV (non-viable refraction, mean K > 55 D, central corneal scars, MCT between 200–300 μm).

The exclusion criteria applied were to have any systemic or ocular pathology and any ocular surgical intervention, including intrastromal rings and corneal collagen cross-linking.

All the eyes were classified according to their Alió et al*.*^15^ calculated a predictive linear regression model of the BSCVA and establishes a grading system of the vision level in four groups according to percentiles. The 25th, 50th, and 75th percentiles for the variable BSCVA were 0.05, 0.19, and 0.40, respectively. Based on these percentiles, 2 groups were formed, each representing a degree of V.L., as follows. Group 1: Patients with BSCVA ≤ 0.05 logMAR units (≥ 0.9 in decimal scale) were classified with no V.L.. Group 2: Patients with BSCVA > 0.05 logMAR units (< 0.9 in decimal scale) were classified with any V.L.

### Patient exam

The patient exam was the same as in our previous, recently published article that analyzed a predictive model for diagnosis of SCK^23^.

Patients were examined by the same trained physician (A.P.R). UCVA and BSCVA were collected with Snellen chart and logMAR chart. Objective refraction by an autorefractometer (KR8900, Topcon, Japan), biomicroscopy (Carl Zeiss Meditec AG, Jena, Germany) and eye fundus were examined.

An analysis of the corneal topography was performed on all patients, under the same dark conditions and in a central diameter of 6 mm of the cornea. Patients with soft contact lenses did not wear them in the examined eye (one for patient) for three weeks, and the gas-permeable rigid lenses for at least five weeks before the test. The topography was performed using a rotating camera Scheimpflug (Pentacam HR, Oculus Optikgeräte, Wetzlar, Germany).

The following Pentacam variables were collected: topographic variables of the anterior corneal surface: the flattest curvature of queratometry (K1), the axis of the flattest curvature of queratometry (K1 Axis), the steepest curvature of queratometry (K2), the axis of the steepest curvature of queratometry (K2 Axis), the mean curvature of queratometry (Km), maximum curvature power on front of the cornea (KMAX), the Q value or coefficient of asphericity that describes the corneal shape factor, or eccentricity of the cornea (Q), the vertical asymmetry index (VAI), the index of height decentration (IHD). Topographic variables of the posterior surface: the flattest curvature (K1), the axis of flattest curvature (K1 Axis), the steepest curvature (K2), the axis of the steepest curvature (K2 Axis), the mean curvature (Km) and the asphericity (Q). Related to pachymetric variables: the central corneal thickness (CCT), the minimum corneal thickness (MCT) with its coordinates (x, y). Related to corneal aberrometry: the root mean square of total aberrations (Total RMS), the root mean square of higher-order aberrations (HOARMS) that were calculated for a 6.0-mm pupil diameter, the astigmatism to 0° (Z2^2^) and 45° (Z2^–2^), the anterior horizontal coma to 0°, the posterior horizontal coma to 0°, the total horizontal corneal coma to 0° (Z3^1^), the anterior vertical coma to 90°, the posterior vertical coma to 90°, the total vertical corneal coma to 90° (Z3^–1^), the trefoil to 0° (Z3^–3^), the trefoil to 30° (Z3^3^), the tetrafoil to 0° (Z4^4^), the tetrafoil to 22.5° (Z4^–4^) and the spherical aberration (Z4^0^). In addition, we collected The Ambrósio relational thickness maximum (ARTmax), the Pachymetry Progression Index (PPI) and the Belin/Ambrósio Enhanced Ectasia Display (BAD-D).

### Statistical analysis

Statistical analysis was performed using the software SPSS Statistics for Windows, version 25.0 (SPSS Inc., Chicago, Ill., USA) and R, version 3.5.1. (R core Team, 2018). The significance level has been p-value < 0.05.

A descriptive analysis of demographic characteristics was carried out in V.L. and no V.L. group. We calculated the frequencies and percentages for qualitative variables and means and standard deviations for quantitative variables. Spearman rank correlation was performed among all tomographic variables and V.L. A relevance model of V.L. was calculated applying a binary logistic regression and Hosmer–Lemeshow test for calibration. V.L. was the dependent variable, and independent variables were included with “forward method”. Then, the R.O.C. (Receiver Operating Characteristic) curve for V.L. model was calculated with the A.U.C. (Area Under the Curve). Sensitivity, specificity, accuracy and precision were estimated with the confusion matrix for model validation.

### Ethical approval

All procedures performed in studies involving human participants were by the ethical standards of the institutional and national research committee and with the 1964 Helsinki declaration and its later amendments or comparable ethical standards. All experiments were performed in accordance with relevant guidelines and regulations. All experimental protocols were approved by Almeria Research Ethics institutional and licensing Committee (CEI/CEIm) located at Torrecárdenas University Hospital and with the following committee’s reference number: 19/2019.

### Informed consent

Written informed consent was obtained from all individual participants included in the study.

## Results

### Demographic characteristics

The study compared 250 patients with keratoconus divided into two groups: no V.L. and V.L. groups; the distribution of them is shown in Table 1. In no V.L. group, there were 99 patients (39.4%) and 151 patients (60.4%) in V.L. group.

**Table 1:** Demographic characteristics of V.L. and no V.L. groups.

	No V.L	V.L
Patients n (%)	99 (39.6)	151 (60.4)
Age (mean ± SD)	33.70 ± 11.53	37.63 ± 15.63
**Eye**		
Right n (%)	47 (47.5)	93 (61.6)
Left n (%)	52 (52.5)	58 (38.4)
**Sex**		
Male n (%)	53 (53.5)	74 (49.1)
Female n (%)	46 (46.5)	77 (50.9)
**Amsler–Krumeich classification**		
SKC n (%)	38 (38.4)	2 (1.3)
Grade 1 n (%)	59 (59.6)	62 (41.1)
Grade 2 n (%)	2 (2)	54 (35.8)
Grade 3 n (%)	0 (0)	19 (12.6)
Grade 4 n (%)	0 (0)	14 (9.3)
**Refraction parameters**		
Sphere (mean ± SD)	− 1.16 ± 2.29	− 4.05 ± 4.71
Cylinder (mean ± SD)	− 1.56 ± 1.20	− 3.48 ± 1.80
Spherical equiv (mean ± SD)	− 1.80 ± 2.12	− 5.46 ± 4.74
**BSCVA (mean ± SD)**		
Decimal scale	0.98 ± 0.04	0.45 ± 0.26
LogMAR scale	0.01 ± 0.19	0.43 ± 0.30

SPSS Statistics for Windows, version 25.0 (SPSS Inc., Chicago, Il, USA).

*V.L.* visual limitation, *SKC* subclinical keratoconus, *BSCVA* best spectacle-corrected visual acuity, *logMAR* logarithm of the minimum angle of resolution.

### Correlation of Pentacam indices with the V.L

In Table 2, the main Pentacam indices and their correlation with V.L. were described. Because of the non-parametric distribution of the variables, Spearman’s rank correlation coefficient was calculated. V.L. was correlated with maximum curvature (*r* = 0.649, R^2^ = 0.421, p < 0.001) and with HOARMS (*r* = 0.625, R^2^ = 0.390, p < 0.001). Corneal vertical coma (*r* = − 0.515, R^2^ = 0.265, p < 0.001) and spherical aberration (*r* = − 0.477, R^2^ = 0.228, p < 0.001) were the two aberrations with the highest correlation values.

**Table 2:** Refractive and topographic parameters and correlation with V.L.

	r (p-value)	R^2^
**Refraction**		
Spherical equivalent	− 0.446 (0.001)	0.199
**Anterior surface topography**		
Kmax	0.649 (0.001)	0.421
Q	− 0.499 (0.001)	0.249
**Posterior suface topography**		
K2	− 0.571 (0.001)	0.326
**Pachymetry**		
MCT	− 0.456 (0.001)	0.208
**Corneal aberrometry**		
HOARMS	0.625 (0.001)	0.390
Corneal vertical coma	− 0.515 (0.001)	0.265
Vertical anterior coma	− 0.503 (0.001)	0.253
Vertical posterior coma	0.453 (0.001)	0.205
Spherical aberration	− 0.477 (0.001)	0.228

SPSS statistics for windows, version 25.0 (SPSS Inc., Chicago, Ill., USA).

*V.L.* visual limitation, *Kmax* maximum curvature, *Q* asphericity, *K2* major posterior curvature, *MCT* minimum corneal thickness, *HOARMS* root mean square high order aberrations.

### Binary logistic regression of V.L. in keratoconus patients

A relevance model of V.L. in keratoconus patients was determined. We have calculated a binary logistic regression model with the program R, version 3.5.1. (R core Team, 2018) with the method of including forward variables. The dependent variable is the presence of V.L. The program calculates the β coefficients and the β exponential corresponding to the Odds Ratio as expressed in Table 3. The Hosmer–Lemeshow test was performed and the result was that the regression model was well-calibrated (p = 0.169). The Hosmer–Lemeshow test is a statistical test for goodness of fit for logistic regression models. It is used frequently in risk prediction models. The test assesses whether or not the observed event rates match expected event rates. Models for which expected and observed event rates are similar are called well-calibrated (p > 0.05).

**Table 3:** Variables of the binary logistic model for V.L. in keratoconus patients.

Variable	β	p-value	OR (exp β)	CI 95% for OR	
Spherical equivalent	− 0.241	0.002	0.786	0.674	0.917
HOARMS	2.458	0.0001	11.681	4.808	28.380
Anterior vertical coma*posterior vertical coma	1.050	0.0001	2.857	1.729	4.723
Spherical aberration	− 2.221	0.003	0.108	0.025	0.473
Constant	− 3.188	0.0001	0.041		

Anterior Vertical Coma*Posterior Vertical Coma, interaction between anterior and posterior vertical coma; Hosmer and Lemeshow test (p = 0.169). R, version 3.5.1. (R core Team, 2018).

*OR* odds ratio, *CI* confidence interval, *RMS* root mean square, *HOA* high order aberrations.

Table 4 shows the matrix table (predicted versus observed cases) for the model. The sensitivity was 91.90%; specificity 83.60%; accuracy 88.94%; and precision 91.17%. Validation of the relevance model was established. The high sensitivity of the model allows to detect early changes in visual acuity in keratoconus patients, and it may be interesting for screening.

**Table 4:** Classification table for logistic regression of the V.L. in keratoconus patients.

	Observed		Total
	V.L	No V.L	
**Predicted**			
V.L	124	12	136
No V.L	11	61	72
Total	135	73	208

Sensitivity = 91.90%; specificity = 83.60%; accuracy = 88.94%; precision = 91.17%; lost cases = 50; R, version 3.5.1. (R core Team, 2018).

*V.L.* visual limitation.

**Figure 1:**
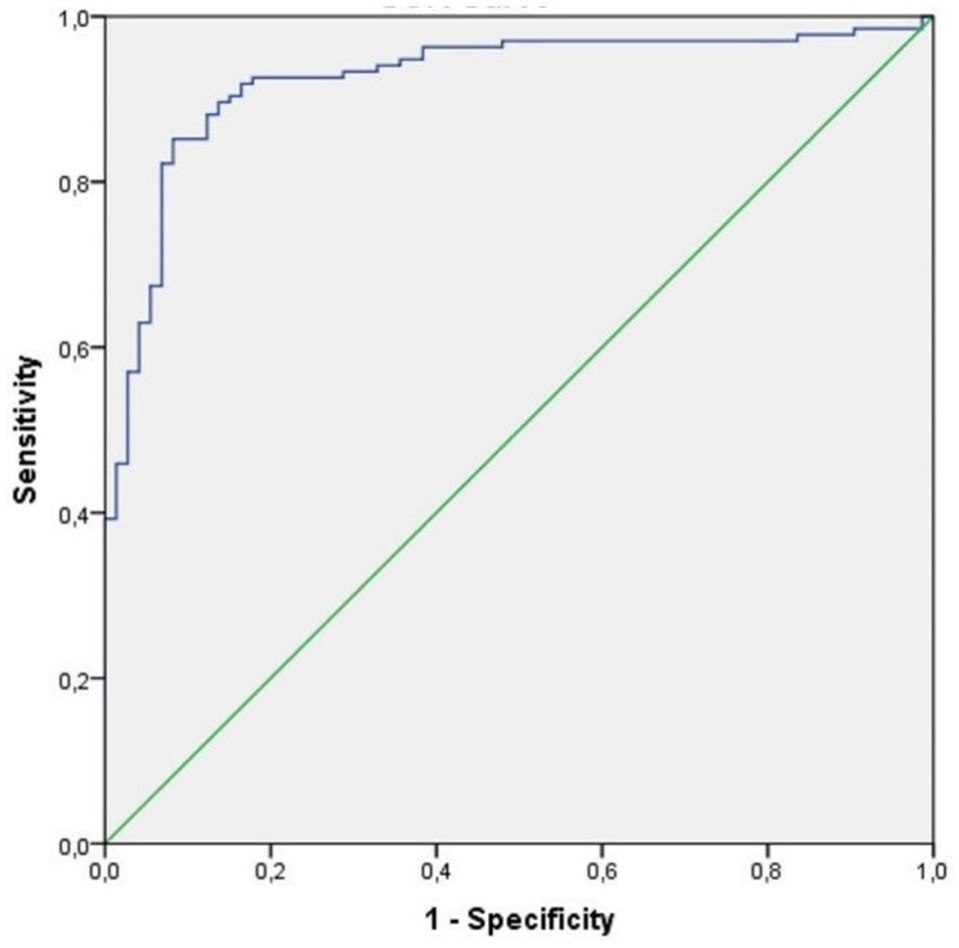
R.O.C. curve for logistic regression for V.L. in keratoconus patients. The A.U.C. of the R.O.C. curve for the binary logistic regression model was 0.924 (CI 95% 0.885–0.963). This means the 92.4% of the predicted “keratoconus with V.L.” would be real “keratoconus with V.L.” if our model would be applied. *V.L.* visual limitation, *A.U.C.* area under the curve, *R.O.C.* receiver operating characteristic. R, version 3.5.1. (R core Team, 2018).

The A.U.C. of the R.O.C. curve (Fig. 1) for the binary logistic regression model was 0.924 (CI 95% 0.885–0.963).

## Discussion

Visual function in patients with keratoconus is the result of integrating the function of different ocular structures. The cornea is the essential structure because of its cone form. In keratoconus patients, irregular astigmatism reduces their quality of vision. This V.L. does not depend on the refractive cause; other factors can be influenced by it such as keratometry, asphericity, intraocular pressure, corneal resistance factor and several high order aberrations^15–17^.

The rotary camera Scheimpflug (Pentacam)^20,24–29^ has been used to the study of keratoconus in clinical practice. Different classifications methods have been used in clinical keratoconus: Amsler–Krumeich^14,29^, Alió and Shabayek^20,25^, KISA % index^30^ or KSS^31^. However, most of them consider topographical morphology of the disease without considering visual function. We used the system grading of V.L. published by Alió et al*.*^15^ based on RETICS classification. Patients with BSCVA ≤ 0.05 logMAR units (≥ 0.9 in decimal scale) were classified as No V.L.. Those with BSCVA > 0.05 logMAR units (< 0.9 in decimal scale) were classified as V.L. Thus, we established two groups: first group with no decreased visual acuity and the second group with early signs of V.L. not spectacles-corrected.

Visual acuity is one of the parameters of Quality of Life (QoL) questionnaires^32^. The Keratoconus Outcomes Research Questionnaire is the only validated keratoconus-specific questionnaire measuring the QoL. Quantity of V.L. can be measured in BSCVA and it is related with different variables: spherical equivalent, mean keratometry, asphericity, intraocular pressure, corneal resistance factor and HOAs^15^.

It is well known that the anterior corneal surface is the essential refractive component of the eye. Anterior corneal aberrations, especially the anterior vertical coma, influence in the visual function of patients with keratoconus^15,19,21,33,34^. However, studies of the posterior surface aberrations are discordant and inconclusive^13,20,24,31,35,36^. Velázquez et al.^37^ published when severe V.L. (0.2 < BSCVA ≤ 0.4 in decimal scale or 6/30 < BSCVA ≤ 6/15 in Snellen chart) appears, anterior corneal topography shows an anterior apex deviation (cone location)^38^. However, in mild keratoconus cases, the posterior corneal surface is determinant for visual function deterioration and early diagnosis^39^. A recent study has published that corneal epithelial thickness would be correlated with BSCVA and discriminates between keratoconus and healthy eyes^40^.

Bayraktar Bilen et al*.*^21^ published that there was correlation between refractive parameters, topographic indices and visual function. There were significant relationships between refractive and visual parameters (p < 0.001). The topographic indices correlated with BSCVA were SRI (surface regularity index) (*r* = 0.670), IAI (irregular astigmatism index) (*r* = 0.660), anterior BFS (Best Fit Sphere) (*r* = 0.586), the steepest keratometric value (K2) (*r* = 0.563) and posterior BFS (Best Fit Sphere) (*r* = 0.551), respectively (p < 0.001 for all). Total RMS and vertical coma correlated better with loss of vision. Esaka et al*.*^22^ found that RMS of corneal elevation (RMSE) (*r* = 0.699) and total coma aberration (*r* = − 0.513) were related with BSCVA (logMAR). In our study, correlations were found among V.L. and spherical equivalent (*r* = − 0.446), Kmax (*r* = 0.649), MCT (*r* = − 0.456), corneal vertical coma (*r* = − 0.515) and spherical aberration (*r* = − 0.477). Bayraktar Bilen et al.^21^ established that the visual quality of patients with keratoconus, measured as BSCVA and contrast sensitivity decreased with parameters such as spherical equivalent, HOARMS, vertical coma, spherical aberration, asphericity, and I-S index. In our investigation, the correlation with V.L. was found with spherical equivalent, Kmax, Q, vertical corneal coma and spherical aberration.

According to previous authors^21,22^, Kmax was the topographic parameter that explains the highest percentage of visual acuity. MCT was found to be a useful parameter for early diagnosis and progression of keratoconus patients. However, it would not modify the visual acuity of these patients. Esaka et al*.*^22^ formulated an equation to calculate BSCVA with RMS of corneal elevation (RMSE) and total coma aberration.

Few papers describe V.L. in keratoconus patients and the parameters that influence the loss of vision. The maximum corneal curvature and the location of the cone are not determining factors in the early visual loss. Initially, the refractive values and the interaction of anterior and posterior corneal surface aberrations are the essential parameters. Scleral^41^ or corneal rigid gas permeable contact lenses^42^ or intrastromal corneal ring segments (ICRS)^43^ are the best solutions in this stage.

There are some limitations to our study. The database has been collected to the southeast of Spain, so there could be a specific genetic component (it is an endemic area of keratoconus) that could vary the predictive value of these models. It could be interesting to increase the sample size in future investigations. Another limitation the discussion has not addressed is that 2nd-order and higher-order aberrations tend to show poor repeatability in keratoconus patients^44^. Furthermore, it is recommended to integrate new parameters such as corneal hysteresis and corneal resistance factor associated to the corneal resistance to deformation. A recent study has demonstrated that there was a significant correlation between epithelial thickness measurements and BCVA^40^.

In conclusion, the V.L. in patients with keratoconus depends on the spherical equivalent (refractive parameter), the interaction between anterior vertical coma and posterior vertical coma and the spherical aberration. All these variables were included in the binary logistic model for V.L. in keratoconus patients. All of them showed a significant correlation with loss of visual acuity in keratoconus patients.

## Data Availability

Castro de Luna, Gracia; Perez-Rueda, Antonio (2020), “VISUAL LIMITATION DATABASE”, Mendeley Data, V1, https://doi.org/10.17632/t34hfym6t7.1.
